# Antitumor effects of cyclin dependent kinase 9 inhibition in esophageal adenocarcinoma

**DOI:** 10.18632/oncotarget.15645

**Published:** 2017-02-23

**Authors:** Zhimin Tong, Devkumar Chatterjee, Defeng Deng, Omkara Veeranki, Alicia Mejia, Jaffer A Ajani, Wayne Hofstetter, Steven Lin, Sushovan Guha, Scott Kopetz, Sunil Krishnan, Dipen Maru

**Affiliations:** ^1^ Department of Pathology, The University of Texas MD Anderson Cancer Center, Houston, TX, 77030, USA; ^2^ Department of Radiation Oncology, The University of Texas MD Anderson Cancer Center, Houston, TX, 77030, USA; ^3^ Department of System Biology, The University of Texas MD Anderson Cancer Center, Houston, TX, 77030, USA; ^4^ Department of Gastrointestinal Medical Oncology, The University of Texas MD Anderson Cancer Center, Houston, TX, 77030, USA; ^5^ Department of Thoracic & Cardiovascular Surgery, The University of Texas MD Anderson Cancer Center, Houston, TX, 77030, USA; ^6^ Department of Gastroenterology, Hepatology and Nutrition, The University of Texas MD Anderson Cancer Center, Houston, TX, 77030, USA

**Keywords:** cyclin dependent kinase 9, esophagus, adenocarcinoma, MCL-1, HIF-1 α

## Abstract

Role of cyclin dependent kinase 9(CDK9) as a potential target in esophageal adenocarcinoma (EAC) is unknown. We investigated CDK9 protein expression in EAC and Barrett's esophagus and role of CDK9 in oncogenic processes of EAC *in vitro* and in murine xenografts. The CDK9 expression was significantly higher in EAC as compared to Barrett's esophagus in patient samples. Stable shCDK9 in SKGT4 reduced proliferation by 37% at day 4, increased apoptosis at 48 hours and induced G1 cell cycle arrest at 48 hours (58.4% vs. 45.8%) compared to controls SKGT4 cells. SKGT4-shCDK9 cell-derived tumors were significantly smaller than control SKGT4-derived tumors in xenografts (72.89mm^3^ vs. 270mm^3^). Pharmaceutical inhibition of CDK9 by Flavopiridol (0.1μm for 48 hours) and CAN508 (20 and 40μm for 72 hours) induced significant reduction in proliferation and 2-fold increase in apoptosis in SKGT4, FLO1 and OE33 cells. In xenograft models, CAN508 (60 mg/kg/dayx10 days) and Flavopiridol (4mg/kg/dayx10 days) caused 50.8% and 63.1% reduction in xenograft tumors as compared to control on post-treatment day 21. Reduction of MCL-1 and phosphorylated RNA polymerase II was observed with transient shCDK9 in SKGT4 cells but not with stable shCDK9. CAN508 (20 and 40 μm) and Flavopiridol (0.1, 0.2 and 0.3 μm) for 4 hours showed reduction in MCL-1 mRNA (84% and 96%) and protein. Mcl-1 overexpression conferred resistance to Flavopiridol (0.2 μm or 0.4 μm for 48 hours) and CAN 508 (20 or 40μm for 72 hours). Chromatin immunoprecipitation demonstrated significant reduction of binding of transcriptional factor HIF-1α to MCL-1 promoter in FLO-1 cells by CDK9 inhibitors.

## INTRODUCTION

Incidence of esophageal adenocarcinoma has increased rapidly in the USA with 16,910 estimated new cases and 15,690 estimated deaths in 2016 [1]. Increase in incidence of adenocarcinoma is primarily responsible for the rise in esophageal cancer in western world [2]. Majority of patients with esophageal adenocarcinoma present with loco-regional (stage II-III) disease. These patients are treated with preoperative chemoradiation followed by esophagogastrectomy. In spite of this aggressive therapy, 5-year survival rate is still low (20-30%). Five year survival rate for metastatic (stage IV) esophageal adenocarcinoma is less than 5%. Major limiting factor for successful implementation of targeted therapy in esophageal adenocarcinoma is low frequency of target specific biomarker alterations and intratumoral heterogeneity [3–6]. Cyclin dependent kinases (CDKs) are the evolutionary conserved ubiquitous serine threonine kinases that play a multifactorial role in regulation of cell cycle and transcription [7, 8]. CDK9, a member of this family, acts predominantly as a transcription regulator by phosphorylating RNA polymerase II (Pol II) carboxyl terminal domain (CTD) and elongation of nascent transcripts [9, 10]. Flavopiridol, a CDK inhibitor with predominant CDK9 inhibitory effects is used in treatment of AIDS and has been tested for its efficacy in clinical trials in lymphoma and other solid tumors [11–13]. CDK9 inhibitor II, CAN508 is an arylazopyrazole compound that inhibits CDK9 with 38-fold selectivity for CDK9/cyclin T over other CDK/cyclin complexes [14] and regulates CDK9 mediated c-MYC and androgen receptor transcription in breast and prostate cancers [15, 16]. More recently, CAN 508 has also demonstrated anti-angiogenesis effects through CDK9-dependent mechanism [17]. Specificity of CAN 508 to CDK9 is partly attributed to the conformational plasticity of CDK9 [18]. The efficacy of these CDK 9 inhibitors in esophageal adenocarcinoma has not been tested. In addition, relevance of CDK9 mediated MCL-1 regulation and mechanism by which CDK9 regulates MCL-1 is not known in esophageal adenocarcinoma, even though CDK9 mediated MCL-1 regulation is one of commonest mechanism by which CDK9 inhibitors have shown anti-tumorogenic effects in other tumors [19, 20]. In this study, we compared CDK9 protein expression in matched samples of Barrett's esophagus and invasive carcinoma from patients with esophageal adenocarcinoma and assessed *in vitro* and *in vivo* effects of genetic downregulation (shCDK9) and pharmaceutical inhibition of CDK9. We also studied mechanism of MCL-1 regulation by CDK9 inhibitors in esophageal adenocarcinoma.

## RESULTS

### Cyclin dependent kinase 9 is overexpressed in esophageal adenocarcinoma and not in Barrett's esophagus

**Figure 1:**
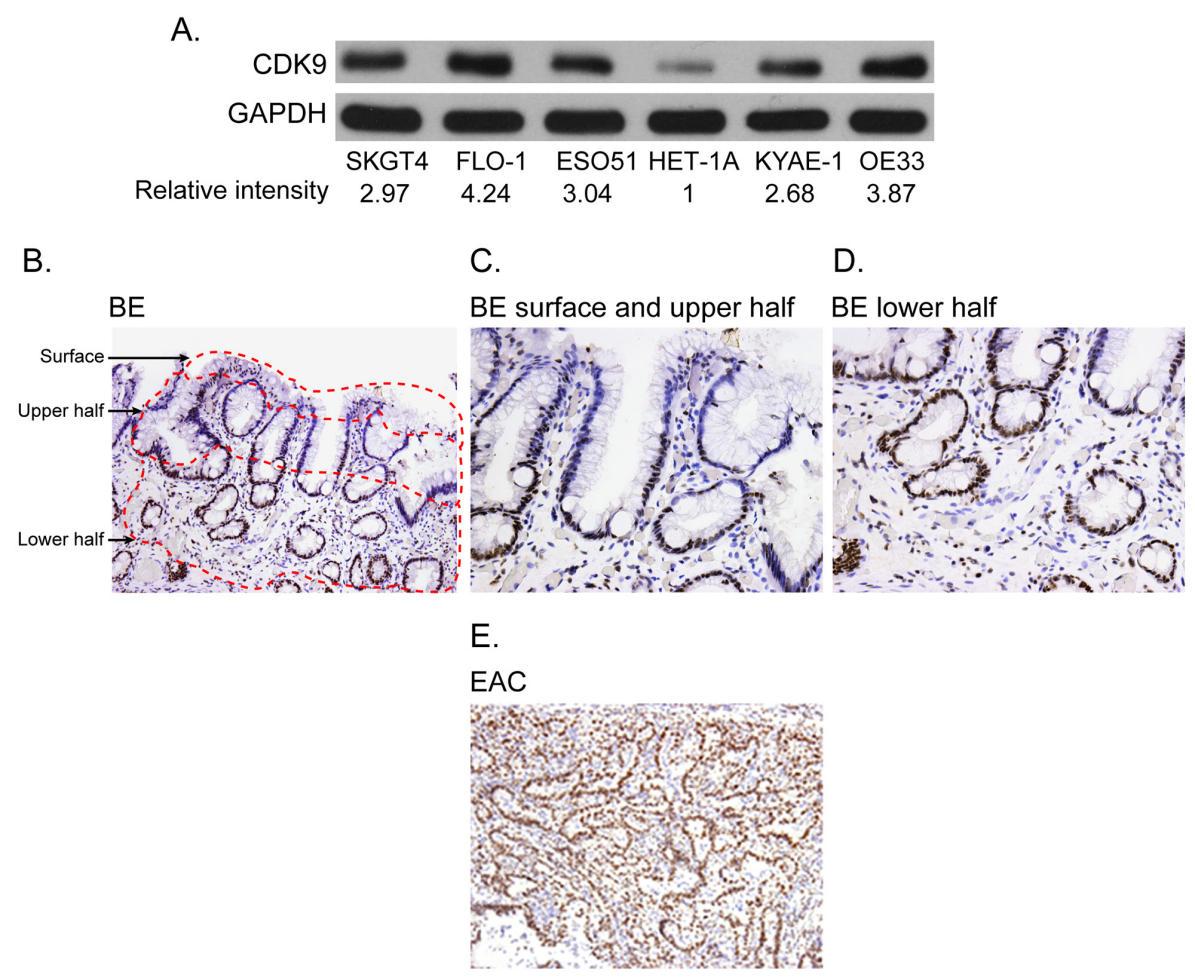
**(A)** Western blot showing CDK9 expression in the esophageal adenocarcinoma and normal squamous epithelial cell lines **(HET-1A)**. Band intensity was measured with Photoshop software. Data is normalized with GAPDH and presented as the relative values to the HET-1A cells. **(B-E)** CDK9 protein expression by immunohistochemistry in matched samples of Barrett's Esophagus (1B, 100X magnification, 1C and 1D 200X magnification) and esophageal adenocarcinoma (1E, 100 X magnification).

All esophageal adenocarcinoma cell lines showed high level of CDK9 protein as compared to a normal esophageal epithelial cell line (Figure 1A). Strong and diffuse expression of CDK9 was observed in more than 90% of invasive adenocarcinoma cells in all tumor samples with minimal to absent staining of the stromal cells. In contrast, CDK9 expression was observed predominantly in the proliferative zone in the base of the crypt of Barrett's esophagus with minimal to absent staining of the surface epithelium (Figure 1B, 1C, 1D). Table 1 shows the quantitative assessment of CDK9 expression in invasive adenocarcinoma and different compartments of Barrett's esophagus. The CDK9 expression was significantly higher in invasive adenocarcinoma (Figure 1E) as compared to CDK9 expression in total (combination of all compartment) Barrett's esophagus and in each compartment of Barrett's esophagus.

**Table 1:** CDK9 staining in matched samples of Barrett's Esophagus and esophageal adenocarcinoma (n=10)

	Percentage of cells with CDK9 staining intensity				p value
		0, mean (range)	1, mean (range)	2, mean (range)	3, mean (range)
**BE surface**	48 (12-90)	17 (0-44)	25 (6-43)	10 (0-48)	<0.01*
**BE upper half**	21 (1-57)	16 (0-35)	36 (22-48)	27 (10-75)	<0.01**
**BE lower half**	3 (0-7)	7 (0-23)	19 (6-34)	73 (31-94)	
**BE total**	23 (4-51)	15 (1-45)	25 (14-34)	37 (16-71)	<0.01***
**Invasive adenocarcinoma**	0	0	10 (0-15)	90 (80-100)	

*p value for for intensity 0, 2, and 3 compared between surface and upper 1/2. p value for staining intensity 1 between surface and upper half of cryptis 0.39

** p value for for intensity 0,1, 2, and 3 compared between upper 1/2 and lower 1/2 of the crypt of BE

*** p value for intensity o,1,2,3 between invasive adenocarcinoma and total BE.

The CDK9 expression in lower half of Barrett's esophagus was significantly higher than the upper half.

### Genetic down-regulation of CDK9 decreases cell proliferation promotes apoptosis and G1 arrest in esophageal adenocarcinoma cells and is anti-tumorigenic in xenografts

**Figure 2:**
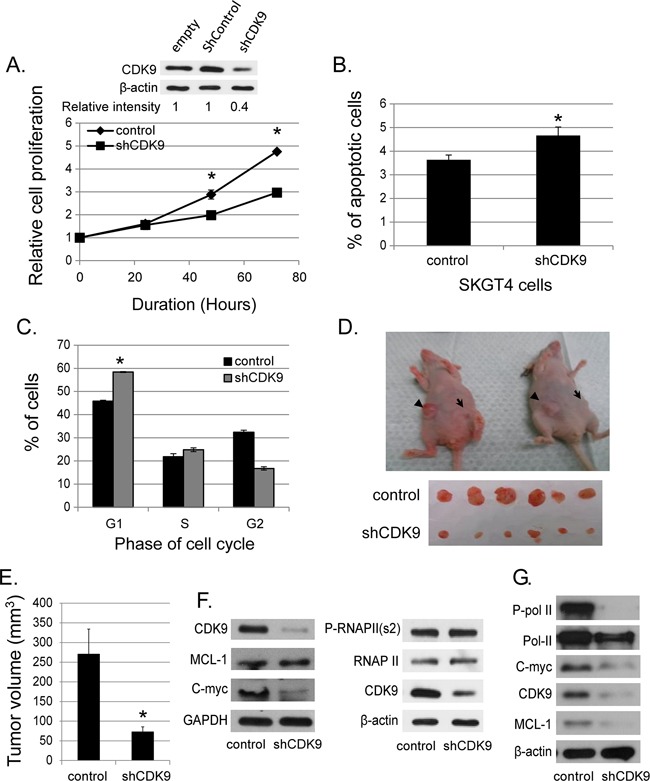
Effects of genetic (shRNA) downregulation of CDK9 **(A)** The down-regulated CDK9 expression in SKGT4 cells by stable shCDK9 was confirmed by western blot (top panel). Effects of stable shCDK9 on cell proliferation in SKGT4. Cell proliferation was measured by MTS assay using the CellTiter Aqueous One Solution Cell Proliferation Assay kit (three separate experiments). **(B)** Effects of stable shCDK9 on the apoptosis of SKGT4 cells (three separate experiments). Cells stained with propidium iodide alone were considered necrotic, whereas cells stained with Annexin V with and without staining with propidium iodide were considered apoptotic. **(C)**. Effects of stable shCDK9 on cell cycle stages in SKGT4 cells (three separate experiments) showing G1 arrest of shCDK9 SKGT4 vs. control. **(D)** Representative photograph of tumor xenografts and **(E)** the volume (means ± SE) of tumor xenografts at end of the experiment with stable shCDK9 and control SKGT4 cells. *represents p-value <0.05 compared to untreated controls. →: xenograft of shCDK9. ►: xenograft of controls. **(F)** Western blot analysis of MCL-1, c-MYC expression and phosphorylation of RNA pol II in stable SKGT-4-shCDK9 and control cells. **(G)** Western blot analysis of MCL-1 and c-MYC expression and phosphorylation of RNA Pol II after transient shCDK9. Cell lysate from cells infected with lenti-shCDK9 for 72 hours were analyzed by western blot with antibodies against to MCL-1, c-MYC or phosphorylated RNA pol II at S2 site (two separate experiments).

We generated stable SKGT4 cells with down-regulated CDK9 expression by transducing lentivirus carrying shCDK9. The down regulation of CDK9 reduced the proliferation of SKGT4 cells by 31.2% at day 3 and 37% at day 4 compared to control cells (p value < 0.01, Figure 2A). ShCDK9 resulted in a significant increase in apoptotic cells (4.6% ± 0.3% vs. 3.6% ± 0.3%, p < 0.05, Figure 2B) and cells in G1 phase at 48 hours (58.4% ± 0.97% vs. 45.8% ± 0.39%, p< 0.01, Figure 2C) compared to the controls SKGT4 cells. In xenograft experiments with genetic downregulation (shCDK9) of SKGT4 and control SKGT4 cells, eleven of 20 mice developed at least one tumor with either parenteral SKGT4 or with shCDK9 SKGT4 cells. There were 16 tumors in 11 mice with parenteral SKGT4 cells (6 mice with 2 tumors, 4 mice with 1 tumor and 1 mouse with no tumor). There were 8 tumors in 11 mice with shCDK9 SKGT4 (1 mouse with 2 tumors and 6 mice with 1 tumor). Four mice with parenteral SKGT4 tumors did not develop tumor with shCDK9 and 1 mouse that developed tumor with shCDK9 SKGT4 did not develop tumor with parenteral SKGT4. Volume of SKGT4-shCDK9 cell-derived tumors was significantly smaller (Figure 2D and 2E) than those from control SKGT4 cells (72.89 ± 12.88 mm^3^ versus 270 ± 64.07 mm^3^, p< 0.01). None of the mice demonstrated signs of morbidity like rapid breathing rate, slow shallow labored breathing, and weight loss, ruffled fur, hunched posture, anorexia and moribund signs like impaired ambulation, muscular atrophy, signs of lethargy, bleeding or CNS disturbances and inability to remain uptight when monitored daily by either staff of department of veterinary medicine or personnel performing experiments. Western blot analysis showed reduction of c-MYC and not of phosphorylated RNA Poll II and MCL-1 with appropriate internal (GAPDH) control and marked reduction of CDK9 in stable shCDK9 SKGT4 cells (Figure 2F). In contrast transient downregulation of CDK9 demonstrated reduction of MCL-1, c-MYC and phosphorylated Poll II (Figure 2G).

### Pharmaceutical inhibition of CDK9 is cytotoxic *in vitro* and has antitumor effects in esophageal adenocarcinoma xenografts

**Figure 3:**
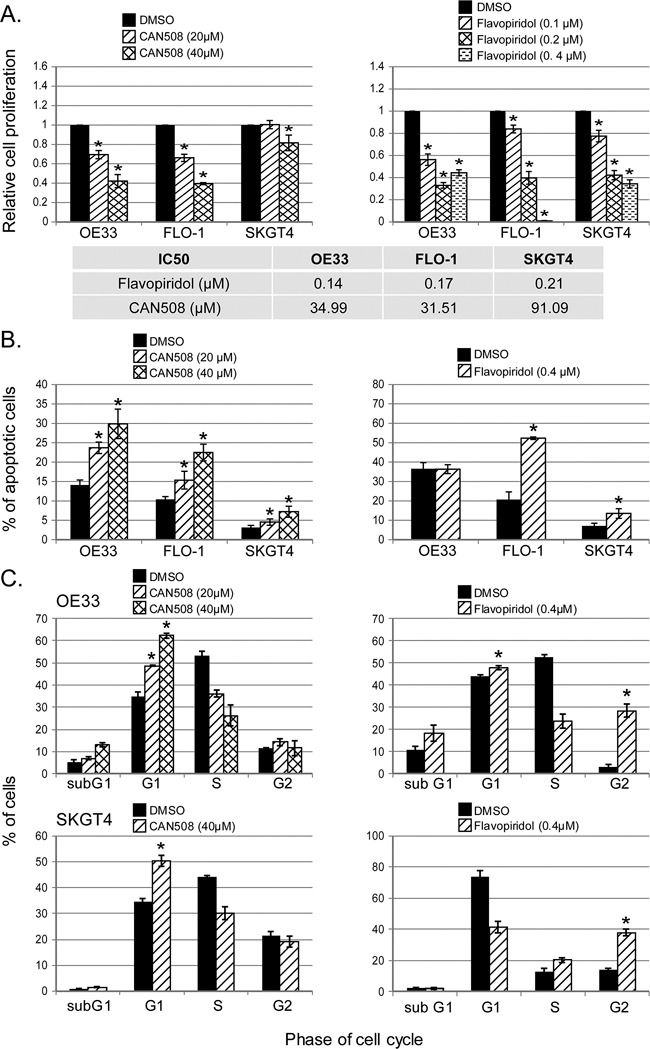
Effects of *pharmaceutical inhibition of CDK9* on cell proliferation, apoptosis and cell cycle Cell proliferation was measured after the treatment of CAN508 for 72 hours (**A**, left panel) or Flavopiridol for 48 hours (**A**, right panel) by MTS assay using Cell Titer Aqueous One Solution Cell Proliferation Assay kit. The apoptosis **(B)** and cell cycle phases **(C)** of esophageal adenocarcinoma cells were determined by flow cytometry. Values are shown as means ± SE of 3 independent experiments. *represents p-value <0.05 compared to untreated controls.

**Figure 4:**
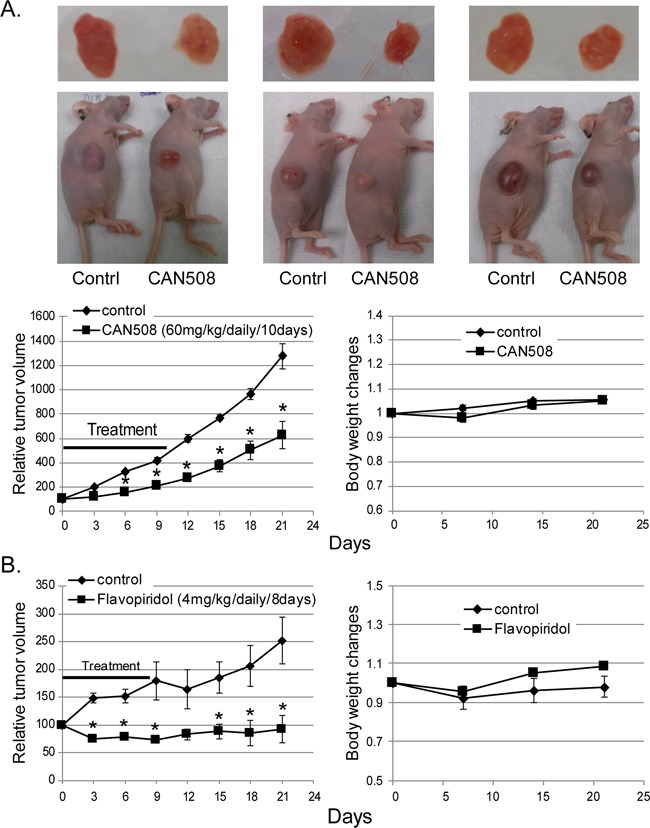
Effects of *pharmaceutical inhibition of CDK9* on EAC cell growth in nude mice Nude mice with subcutaneously injected with 4×10^6^ of FLO-1 cells. Mice bearing established xenograft were then treated with a dose of 60 mg/kg CAN508 by once a day intraperitoneal injection for 10 days **(A)** or a dose of 4 mg/kg of Flavopiridol by once a day intraperitoneal injection for 8 days **(B)**. Tumor growth was measured by tumor volume. Data are presented as the percentage of tumor growth. *represents p-value < 0.01 compared to control group. Body weight was measured once a week and data is shown relative to the body weight at day of treatment starting.

CAN508 and Flavopiridol significantly reduced cell proliferation in a dose dependent manner in all three esophageal adenocarcinoma cell lines *in vitro* (Figure 3A). Significant inhibition of SKGT4 cell proliferation was detected with 72 hours treatment with a dose of 40 μm of CAN508 while a dose of 20 μm of CAN508 was sufficient to inhibit the proliferation of OE33 and FLO-1 cells (p <0.05). A dose of 0.1 μm for 48 hours was sufficientfor Flavopiridol to inhibit the proliferation of all three esophageal adenocarcinoma cell lines. CAN508 (40μM for 72 hours) also increased apoptosis by 2 fold in all three esophageal adenocarcinoma cells compared to untreated controls. Flavopiridol (0.4 μm for 48 hours) increased apoptosis in FLO-1 and SKGT4 cells as compared to control (Figure 3B). CAN508 treatment for 72 hours led to the accumulation of cells in G1 phase (Figure 3C) and Flavopiridol treatment for 48 hours led to accumulation of cells in G2 phase. In xenograft models, both CAN508 and Flavopiridol caused reduction of tumor growth starting from post-treatment day three with 50.83% reduction with CAN508 (Figure 4A, p<0.01 compared to control) and 63.1% reduction with Flavopiridol (Figure 4B, p<0.001 compared to control) on post-treatment day 21. There were no significant signs of toxicity throughout the treatment period as monitored by bodyweights (Figure 4A and 4B) and other signs of toxicity including rapid breathing rate, slow shallow labored breathing, abdominal distension, ruffled fur, hunched posture, anorexia and moribund signs like impaired ambulation, muscular atrophy, signs of lethargy, bleeding or CNS disturbances and inability to remain uptight when monitored daily by either staff of department of veterinary medicine or personnel performing experiments.

### Pharmaceutical inhibition of CDK9 transcriptionally regulates MCL-1 mRNA and does not modify proteosomal degradation of MCL-1 in esophageal adenocarcinoma *in vitro*

**Figure 5:**
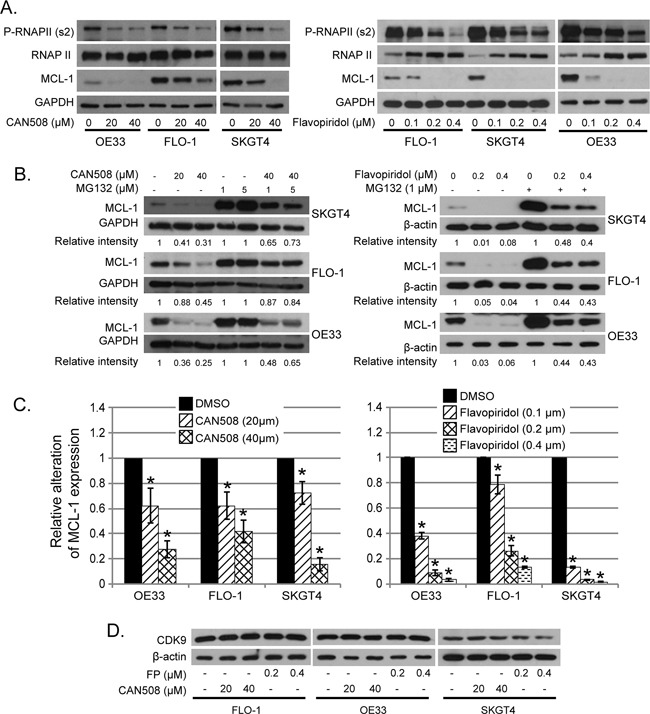
Effects of pharmaceutical inhibition of CDK9 on RNA Pol II phosphorylation and MCL-1 expression **(A)** Cells were treated with CAN508 or Flavopiridol for 4 hours. The phosphorylation of RNA Poll II and the expression of MCL-1 were examined by western blot. **(B)** Cells were treated with CAN508 or Flavopiridol for 4 hours after pretreatment with or without MG123 for 1 hour. Expression of MCL-1 was then detected by western blot. The band intensity was measured with Photoshop software. The data was normalized with host genes (GAPDH or β-actin) and presented as the relative values to corresponding controls. **(C)** The mRNA levels of MCL-1 were measured by Q-PCR after treatment of CAN508 (left panel) or Flavopiridol (right panel) for 4 hours at indicated dose. Value are shown as means ± SE of three independent experiments. *represents p-value <0.05 compared to untreated controls. **(D)**. Cells were treated with CAN508 or Flavopiridol for 4 hours. The phosphorylation of RNAPII and the expression of c-MYC and CDK9 were examined by western blot.

CAN508 (20 and 40 μm) treatment and Flavo-piridol treatment (0.1, 0.2 and 0.3 μm) for 4 hours caused dramatic reduction of phosphorylation at ser2 of Pol II carboxy terminal domain (Poll II CTD) and MCL-1 protein (Figure 5A). Reduction of phosphorylation at ser2 of Poll II CTD and MCL-1 by CAN508 and Flavopiridol was in dose dependent manner and lasted for at least 16 hours (data not shown). Ubiquitin dependent proteosomal degradation is a well-established mechanism of MCL-1 reduction in cells [21, 22]. MG-132 is an inhibitor of ubiquitin dependent proteosomal degradation. In our experiments, MCL-1 level was significantly higher in cells treated with CDK9 inhibitor and MG132 as compared to cells treated with only CDK9 inhibitor (p<0.05). However, the MCL-1 level is significantly lower in cells treated with MG132 and CDK9 inhibitor as compared to MG132 alone (p<0.05). This indicates that proteosomal degradation inhibition partly rescues the MCL-1 and rest of reduction in MCL-1 protein is likely to be secondary to reduced transcription (RNA level). (Figure 5B). In contrast, *MCL-1* mRNA expression was reduced by 72.5% ± 6.8% in OE33 cells, by 58% ± 9.1% in FLO-1 cells and by 84.3% ± 5.5% in SKGT4 cells after treatment with CAN508 (40 μm) for 4 hours as compared to the expression in untreated cells. (p value < 0.05, Figure 5C, left panel). Treatment with Flavopiridol (0.4 μM, 4 hours) resulted in reduction of MCL-1 mRNA expression by 96.8% ± 1% in OE33 cells, 87.1% ± 0.7% in FLO-1 cells and 98.5% ± 0.5% in SKT4 cells (Figure 5C, right panel). These findings indicate that Flavopiridol and CAN508 regulates MCL-1 predominantly by inhibiting MCL-1 transcription and less so by enhancing proteosomal degradation. CAN508 treatment with 40 μm for 4 hours showed reduction in c-MYC in OE 33 cells, while no reduction in c-MYC was observed after treatment with CAN 508 in Flo-1 and SKGT4 cells and with lower dose (20 μm) OE 33 cells. Treatment with Flavopiridol (0.2 μm and 0.4 μm) for 4 hours decreased c-MYC in OE33 and SKGT4. In Flo-1 cells only higher dose (0.4 μm) of Flavopiridol decreased c-MYC (Figure 5D). Flavopiridol and CAN508 are competitive inhibitors of CDK9/positive transcription elongation factor (p-TEFB) and do not affect CDK9 levels in the tumor cells as shown in Figure 5D.

### Pharmaceutical inhibition of CDK9 regulates MCL-1 by inhibiting promoter binding of HIF1-alpha in esophageal adenocarcinoma cells

**Figure 6:**
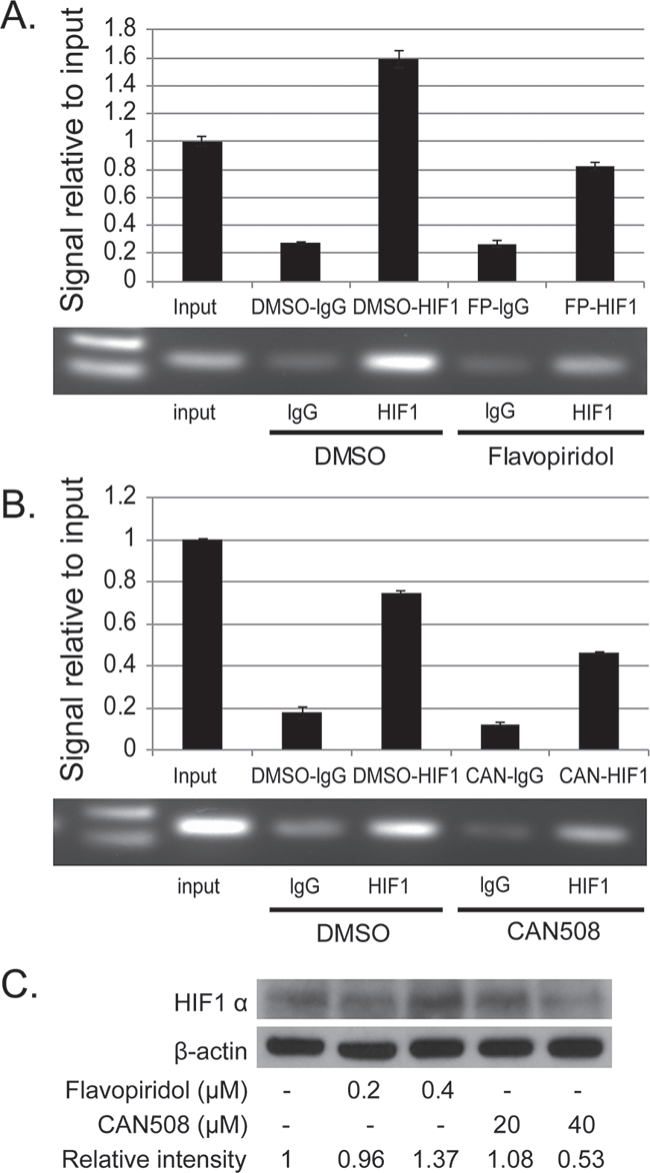
Effects of *pharmaceutical inhibition of CDK9* on the binding of HIF-1α to MCL-1 promoter The binding of HIF-1α to MCL-1 promoter region was evaluated by ChIP assay in FLO-1 cells treated with 40μM of CAN 508 **(A)** or 0.4 μM of Flavopiridol **(B)** for 4 hours as described in Materials and Methods. Q-PCR results show the mean of triplicate for each treatment from a typical experiment; bars, ±SE. Similar results were observed in two independent experiments. **(C)** Western blot analysis of HIF1α expression after the treatment of CDK9 inhibitors for 4 hours. The band intensity was measured with Photoshop software. The data was normalized with β-actin and presented as the relative values to controls.

CAN508 (40 μM, 4h) and Flavopiridol (0.4 μM, 4h) significantly reduced signal of MCL-1 promoter bound to HIF-1α antibody as compared to control (DMSO) with very low signal for non-specific binding to RbIgG. Input DNA levels are unrelated to binding of DNA to protein and are primarily used a control (Figure 6A and 6B). No change in HIF1-alpha protein was observed after treatment with Flavopiridol (0.2 and 0.4 μM, 4h) and CAN 508 (20 μM, 4h) and minimal decrease was observed with CAN 508 (40 μM, 4h, Figure 6C). These findings indicate CDK9 inhibitors reduce binding of transcription factor HIF-1 α to −1051 to −901bp region of MCL-1 promoter.

### Over expression of MCL-1 reduces the cytopathic effects of CDK9 inhibitors

Cells with MCL-1 overexpression had very high levels of MCL-1 compared to MCL-1 expression in control cells (Supplementary Figure 1A). As expected, MCL-1 overexpression led to a reduction of CDK9 inhibitors cytotoxicity in esophageal adenocarcinoma cells. All three cell lines with MCL-1 overexpression were significantly more resistant to 40 μm of CAN508 than the control cells (p< 0.05, Supplementary Figure 1B). However, only FLO-1 and SKGT4 cells with MCL-1 overexpression were significantly more resistant to Flavopiridol than control cells (Supplementary Figure 1C).

## DISCUSSION

In this study, we have demonstrated preclinical *in vitro* and *in vivo* efficacy of CDK9 inhibition in esophageal adenocarcinoma, by genetic downregulation and pharmaceutical inhibition. shCDK9 and pharmaceutical inhibition by an established clinically used CDK9 inhibitor (Flavopiridol) and highly specific CDK 9 inhibitor (CAN508) demonstrated reduction in cell proliferation, increase in apoptosis and G1 or G2 cell cycle arrest. Similarity in cytotoxic effects between genetic downregulation and both CDK9 inhibitors support CDK9 as an important therapeutic target in esophageal adenocarcinoma. Flavopiridol and CAN508 had similar degree of dose dependent anti-apoptotic effects in 2 esophageal adenocarcinoma cell lines and dose dependent anti-proliferative effects in all 3 esophageal adenocarcinoma cells lines. Flavopiridol demonstrated higher anti-proliferative effects in SKGT4 cells as compared to CAN 508. In contrast CAN 508 demonstrated higher anti-apoptotic effects in OE33 as compared to Flavopiridol. SKGT4 cells are derived from low T stage (T2) well differentiated esophageal adenocarcinoma while FLO-1 cells are derived from stage III/IV esophageal adenocarcinoma and OE33 cells are derived from stage III poorly differentiated adenocarcinoma. These differences in histology and stage of the disease may explain different biology of the tumor cells which is reflected in sensitivity to CDK9 inhibitors. The difference in cell cycle arrest (G1 vs. G2) with Flavopiridol and CAN 508 is possibly due to effects of Flavopiridol on CDKS other than CDK9 and less specificity towards CDK9 as compared CAN 508 as shCDK9 also demonstrated G1 arrest. In xenograft experiments, difference in rate of xenograft volume change between Flavopiridol and CAN508 is possibly due to different strains of mice as number and type of cells injected were similar. In both experiments, xenograft tumor growth was significantly inhibited by the CDK9 inhibitors compared to control from day 3 (Flavopiridol) and day 6 (CAN508) to the end of experiment indicating efficacy of both inhibitors in controlling tumor growth in esophageal adenocarcinoma xenografts.

In the present study, both CDK9 inhibitors (doses lower than calculated IC_50_) and transient shCDK9 downregulated p-Pol II and MCL-1 while stable shCDK9 did not down regulate p-Pol II and MCL-1. This is likely due to the phosphorylation of Pol II and activation of MCL-1 by alternate pathways in stable shCDK9 cells because of the irreversible effects of stable shCDK9 as compared to reversible effects of transient shCDK9 and CDK9 inhibitors. The difference in effects of type (stable vs. transient) of genetic downregulation of CDK9 on critical downstream targets of CDK9 (p-Pol II and MCL-1) is new and additional work with different types of genetic downregulation in esophageal adenocarcinoma and other solid tumor cell lines will provide more insights in understanding mechanism of action of CDK9 and identifying appropriate target for the CDK9 inhibition in esophageal adenocarcinoma. Transient downregulation (as compared to stable downregulation) is functionally more similar to treatment with CDK9 inhibitors due to temporary effects of CDK9 inhibition. Similarities in effects of transient shCDK9 and CDK9 inhibitors on p-Poll II (surrogate of CDK9 activity) and MCL-1 support our conclusion that cytotoxic effects of CAN 508 and Flavopiridol are at least partly mediated by CDK9 and MCL-1 is a potential target of these inhibitors in esophageal adenocarcinoma cells. This conclusion is further supported by reduction of efficacy of Flavopiridol and CAN 508 *in vitro* with MCL-1 upregulation/overexpression in at least 2 esophageal adenocarcinoma cells.

For both CDK9 inhibitors, MCL-1 downregulation was dose dependent and associated with decrease in phosphorylation of Poll II at ser 2. These along with no increase in ubiquitin mediated proteosomal degradation by pharmaceutical CDK9 inhibitors, suggest that CDK9 transcriptionally regulates MCL-1 in esophageal adenocarcinoma. A previous study has shown that HIF-1α expression is significantly higher in esophageal adenocarcinoma as compared to Barrett's esophagus [23], similar to what is observed with CDK 9 in our study. However, role of HIF-1α in critical cellular processes of esophageal adenocarcinoma is unknown. In this study, we for the first time demonstrate that MCL-1 regulation by CDK9 inhibitor is mediated by downregulation of binding of transcription factor HIF-1α to MCL-1 promoter. Interplay of MCL-1 and HIF-1α have shown to be variable in different tissues and cancers. In hepatoma cells, HIF-1α induced MCL-1 upregulation is anti-apoptotic [24] while in small cell lung cancer cell lines, hypoxia induced MCL-1 downregulation is independent of HIF-1α [25]. It will be interesting to study whether the CDK9 inhibitor induced HIF-1α mediated MCL-1 downregulation is dependent on the hypoxic injury and what are the end results of HIF-1α mediated MCL-1 downregulation on other processes critical to malignant phenotype like angiogenesis. Prior studies have shown that CDK9 mediated transcription regulation of MYC is important in therapy resistance in breast cancer and disease maintenance in hepatocellular carcinoma [15, 26, 27]. Our findings show that MYC is downregulated with shCDK9 in esophageal adenocarcinoma while MYC downregulation by the CDK9 inhibitors is not consistent across three *esophageal adenocarcinoma* cell lines. As focus of this study was to assess the efficacy and identify a target of pharmaceutical inhibitors of CDK9 in esophageal adenocarcinoma and MYC did not show consistent alterations after CDK9 inhibitor treatment, we chose to study MCL-1 instead of MYC as the CDK9 target in this study.

Progress in targeted therapy in esophageal adenocarcinoma has been slow primarily due to failure of conventional markers in other gastrointestinal adenocarcinoma (KRAS, EGFR, PTEN, PIK3CA, and c-MET) [28] to identify patients with esophageal adenocarcinoma who would respond to a targeted therapy. In addition, lower frequency of biomarkers like HER2-Neu in esophageal adenocarcinoma has provided limited benefit of Trastuzumab in a small group of patients [29]. Cyclin dependent kinase 9 inhibition is potentially a good therapeutic strategy as CDK9 is diffusely overexpressed in esophageal adenocarcinoma cells as compared to Barrett's esophagus. The CDK9 overexpression is related to the proliferative nature of the cells as higher expression was observed in adenocarcinoma cells and lower half of the crypts (proliferative compartment rich in stem cells) of Barrett's esophagus as compared to upper half of the crypts of Barrett's esophagus. The higher expression of CDK9 in actively proliferating cells also supports use of CDK9 inhibitors in combination with chemotherapy agents as both the agents are likely to be effective in rapidly dividing esophageal adenocarcinoma cells. The CDK9 inhibitors used in this study were selected based on their efficacy in other tumors and specificity to CDK9, to study the relevance of CDK9 inhibition in esophageal adenocarcinoma. Our findings support further exploration of newer CDK9 inhibitors which are more specific and likely to cause lower toxicity with therapeutic doses in patients with esophageal adenocarcinoma compared to the previously used CDK9 inhibitors. In addition, role of MCL-1 in selecting patients whose tumor is more likely to respond to these CDK9 inhibitors with or without chemotherapy and radiation need to be studied in esophageal adenocarcinoma.

In summary, findings in this study demonstrate significant *in vitro* and *in vivo* efficacy of CDK9 inhibition in esophageal adenocarcinoma and identify that CDK9 regulates MCL-1 transcription by inhibiting binding of HIF-1alpha to MCL-1 promoter. With limited options of targeted therapy in esophageal adenocarcinoma, exploration of CDK9 inhibitors as therapeutic agents in patients with esophageal adenocarcinoma is warranted.

## MATERIALs AND METHODS

### Histopathology review and immunohistochemical analysis

Patient population comprised of 9 men and 1 woman with an average age of 73 years (range 54-90 years). All patients underwent standard pretreatment staging which included esophagogastroduodenoscopy with endoscopic ultrasound (EUS) and biopsy of tumor and Barrett's esophagus and CT or PET-CT of chest and abdomen. All patients had clinical stage I disease with tumor limited to mucosa or submucosa with negative lymph nodes by EUS and fine needle aspiration. Long segment (length of segment more than 3 cm long) Barrett's esophagus was found in 7 patients and short segment (length of segment less than 3 cm) Barrett's esophagus was found in 3 patients. Three patients had biopsy proven high grade dysplasia away from tumor, 5 patients had low grade dysplasia away from tumor and 2 patients had non dysplastic Barrett's esophagus away from tumor. Seven patients underwent endoscopic mucosal resection and three patients underwent esophagogastrectomy without preoperative chemoradiotherapy. One of ten patients died of metastatic disease, one patient died because of sepsis unrelated to esophageal adenocarcinoma and 8 patients were alive at the time of last follow up.

Formalin fixed paraffin embedded tissue sections from matched Barrett's esophagus and adenocarcinoma from surgically or endoscopically resected specimens were stained for Hematoxylin and Eosin (H&E) and CDK9 protein expression by immunohistochemistry. H&E slides were reviewed by an expert gastrointestinal pathologist (DMM) and standard histopathology diagnostic criteria were applied to confirm diagnoses of invasive adenocarcinoma and Barrett's esophagus with and without dysplasia [30]. Invasive adenocarcinoma was diagnosed when stromal invasion beyond basement membrane was identified in the H & E stained sections. Barrett's esophagus was diagnosed as presence of intestinal metaplasia (presence of goblet cell). Presence and grading of dysplasia in Barrett's esophagus were assessed based on absence of epithelial maturation on the surface, architectural complexity, increase in nuclear/cytoplasmic ratio and presence of surface mitosis in the metaplastic cells [31]. For immunohistochemistry, tissue sections were deparaffinized and antigen retrieval was performed with citrate buffer for 15 min at 100°C. Anti-CDK9 antibody (rabbit monoclonal antibody, Cell Signaling Technology) was then added for 16 hours at 4°C. Endogenous peroxidase activity was blocked by 3% hydrogen peroxidase. The immunoreative protein was visualized by Ventana DAB detection system (Dako, Carpenteria, CA). CDK9 nuclear staining intensity was assessed in surface, upper half and lower half (proliferative compartment) of the crypts of non-dysplastic Barrett's esophagus (intestinal metaplasia) and in the invasive adenocarcinoma cells. Percentage of cells with nuclear intensity 0, 1, 2 or 3 were manually counted in each compartment of Barrett's esophagus and invasive adenocarcinoma cells in 10 fields at 200X magnification by a gastrointestinal pathologist (Supplementary Figure 2).

### Cell lines and cell culture

Esophageal adenocarcinoma cells OE33, FLO-1 and SKGT4 were purchased from Sigma-Aldrich (St. Louis, MO). ESO51 and KYAE-1 cells were obtained from **Culture Collections** (Public Health England, UK). OE33, ESO51 cells were maintained in a RPMI medium containing 2 mM of L-Glutamine, 10% of fetal bovine serum (FBS), 100 units/ml penicillin and 100 μg/ml of streptomycin. KYAE-1 cells were maintained in a medium of RPMI + Hams F12 (1:1) and FLO-1 and SKGT4 cells were maintained in a DMEM medium containing 10% of FBS, 100 units/ml penicillin and 100 μg/ml of streptomycin. 293 FT cells were obtained from Invitrogen (Carlsbad, CA) and maintained in a DMEM medium supplemented with 10 % FBS and 500μg/ml G418. Normal esophageal epithelial cells, HET-1A were provided by Dr. Xu (MD Anderson Cancer Center, Houston, TX) and maintained in a KSF medium (Lonza Walkersville Inc., Walkersville, MD). Cells were maintained in a 5% CO_2_ atmosphere at 37°C and passaged at 80% confluence using 1 mM EDTA-0.025% trypsin for 3 to 5 minutes. All cell lines were authenticated by cell line validation core facility of UT M.D. Anderson Cancer Center.

### Western blot

Proteins from cell lysates were separated on 8% or 10% SDS-PAGE gel. The separated proteins were electrophoretically transferred to PVDF transfer membranes (GE Healthcare Life Sciences, Pittsburgh, PA) and incubated with a blocking solution, 5% dry milk in TBST [25 mmol/L Tris-HCl (pH 7.6), 200 mmol/L NaCl, and 0.1% Tween 20], for 1 h at room temperature. Target protein levels were measured by immunoblotting with antibodies of CDK9 (rabbit monoclonal, Cell Signaling Technology), MCL-1, c-Myc (Santa Cruz Biotechnology, Inc., Santa Cruz, CA), RNA pol II (p Ser2), RNA pol II (Novus biological, Littleton, CO) and HIF-1alpha (ThermoFisher Scientific, Waltham, MA). Blots were washed thrice for 15 min each time at room temperature with TBST and then incubated for 1 h with secondary anti-mouse or anti-rabbit peroxidase-linked antibodies (GE Healthcare Life Sciences, Pittsburgh, PA) in a blocking solution. Blots were then washed (3 × 15 min). Bands were visualized by enhanced chemiluminescence (GE Healthcare Life Sciences, Pittsburgh, PA). All experiments were performed in triplicates.

### Quantitative real-time PCR

Total RNA was isolated from cells using the RNeasy mini kit according to the manufacturer's protocol (Qiagen, Valencia, CA). 0.5 μg of RNA was then reverse-transcribed to cDNA using SuperScript II Reverse Transcriptase (Invitrogen, Carlsbad, CA). Quantitative real-time PCR was performed using SYBER Green mix on a BioRed instrument. PCR primers were designed using primer3 program according to DNA sequence of MCL-1. The quantitative real-time PCR for each treatment was performed in triplicate. *Ct* value was obtained by BioRed iCycler data analysis software. The *Ct* value of GAPDH was subtracted from that of the interested gene to obtain a Δ*Ct* value. The Δ*Ct* value of controls was subtracted from the Δ*Ct* value of each sample to obtain a ΔΔ*Ct* value. The gene expression level relative to the controls was expressed as 2^−ΔΔ*Ct*^. All experiments were performed in triplicates.

### CDK9-shRNA esophageal adenocarcinoma cells and pharmaceutical inhibitors of CDK9

To produce lentivirus that expresses shCDK9, we cotransfected pLKO-shCDK9 (Sigma-Aldrich, St. Louis, MO), or control vectors with their packaging and envelope plasmids into 293FT cells using lipofectamine 2000 reagent according to the manufacturer's instructions (Invitrogen, Carlsbad, CA). Forty eight hours later, viral supernatant was collected after centrifugation at 3000 rpm for 15min. For transduction with lentivirus, cells were infected with 2x diluted virus media containing 6 μg/ml of polybrene for 16 hours. Cells with stably down- regulated CDK9 expression by shCDK9 were selected by incubation in a medium containing purimycin for at least 2 weeks. The expression of target proteins was confirmed by western blot. For transient down regulation of CDK9, cells were harvested for western blot after 72 hours infection with lenti-shCDK9.

Pharmaceutical inhibitors of CDK9, CAN508 and Flavopiridol were purchased from EMD Millipore (Temecula, CA) and Cayman Chemical (Ann Arbor, MI), respectively. Both were dissolved in DMSO before their use *in vitro* or in xenografts.

### Generation of stable MCL-1 overexpression in esophageal adenocarcinoma cells

Human MCL-1 cDNA was released from pCMV-SPORT6 (OriGene technologies Inc., Rockville, MD) with EcoRI and subcloned into the lentiviral vector pCDH-VMV-MCS-EF1-Puro between EcoRI to create phMCL-1. Identity and orientation of this construct were confirmed by DNA sequencing (DNA core facility, MD Anderson Cancer center, Houston, TX). To produce lentivirus that overexpresses MCL-1, we cotransfected phMCL-1 or control vectors with their packaging and envelope plasmids into 293FT cells using lipofectamine 2000 reagent according to the manufacturer's instructions (Invitrogen, Carlsbad, CA). Forty eight hours later, viral supernatant was collected after centrifuged at 3000 rpm for 15min. For transduction with lentivirus, cells were infected with 2x diluted virus media containing 6 μg/ml of polybrene for 16 hours. Cells with stable expression of MCL-1 were selected by incubation in a medium containing purimycin for at least 2 weeks. Expression of target proteins was confirmed by western blot.

### Cell apoptosis assay and cell cycle analysis

For apoptosis assay, esophageal adenocarcinoma cellsafter shCDK9 ortreatment with CAN508 for 72 hours or Flavopiridol for 48 hours were harvested, washed with cold PBS, resuspended in a solution containing 5 μl of recombinant Annexin V-FITC (BD Biosciences, San Jose, CA) and 5 μg/ml of propidium iodide, and incubated for 15 minutes. For cell cycle, cells treated with CAN508 for 72 hours or Flavopiridol for 48 hours were fixed and stained with propidium iodide. Apoptotic cells and cell cycle were then analyzed by flow cytometer at MD Anderson Cancer Center DNA analysis core facility (Houston, TX). Cells stained with propidium iodide alone were considered necrotic, whereas cells stained with Annexin V (Annexin V+ cells) with and without propidium iodide were considered apoptotic.

### Chromatin immunoprecipitation assay (ChIP)

We performed ChIP assay to confirm that the reduction of MCL-1 expression by CDK9 inhibitors is at the transcriptional level and to determine whether CDK9 inhibitors affect the binding of transcriptional factors such as HIF-1α to MCL-1 promoter. HIF-1α has been reported to play important roles in regulation of MCL-1 expression [32, 33]; although HIF-1α mediated regulation of MCL-1 has not been studied in esophageal adenocarcinoma. ChIP assay was performed using Pierce™ agarose ChIP kit (Thermo Scientific, Rockford, IL). Briefly, FLO-1 cells were treated with 0.4 μm of Flavopiridol or 40 μm CAN508 II for 4 hours and fixed by 1% formaldehyde solution to cross-link DNA and protein. The chromatin was then digested with Micrococcal Nuclease to obtain chromatin fragments ranging in size from 200 to 1000 base pairs. 10% of chromatin fragments were used as input DNA. The immunoprecipitation was performed using either 1 μg of anti-HIF-1α antibody or lgG control. The immunoprecipitated DNA was then quantitated using real-time PCR. The specific primers for MCL-1 promoter (Forward: 5′-AGGTCACTTGAGGCCATGAG-3′, Reverse: 5′-CACGTTCAGACGATTCGGTA-3′) were used as previously reported [32]. These primers cover −1051 to −901bp region of MCL-1 promoter. The enrichment of targeted genomic regions was assessed relative to the input DNA. ChIP assay was run twice with both inhibitors and Q-PCR in all 4 ChIP experiments were run in triplicates.

### Esophageal adenocarcinoma xenografts studies

All experiments involving mice were conducted according to animalexperimental protocol that has been approved by the Institutional Animal Care and Use Committee (IACUC) at The University of Texas MD Anderson Cancer Center. To assess the *in vivo* effects of genetic downregulation of CDK9 (shCDK9), 2×10^6^ of SKGT4-shCDK9 and control SKGT4 cells were injected subcutaneously in abdominal wall of 4 weeks-old female nude mice. Total 20 mice were injected with 4 injections per mice. The injection sites included right upper and left lower quadrants for parenteral SKGT4 cells and left upper and right lower quadrants for shCDK9 SKGT4 cells. Distance between injection sites was at least 3 cm. The volume of xenograft tumor was measured every three days until 7 weeks using a digital caliper. All mice were euthanized and tumor tissues were harvested at the end of 7^th^ week. The tumor volume was calculated as per the institutional IACUC recommended protocol as (W^2^ x L)/2 (in which W=smallest diameter of tumor, L=largest diameter of tumor). Outer edge of the tumor was more than 2 cm away from nearest tumor and no tumor was identified in the tissue between tumors on inspection and palpation.

Efficacy of CAN508 and Flavopiridol in xenografts of esophageal adenocarcinoma cells were studied in two separate experiments. One experiment compared efficacy of CAN508 (60 mg/kg given daily intraperitoneally for 10 days) with control and other experiment compared efficacy of Flavopiridol (4 mg/kg, given daily intraperitoneally for 8 days) with control. FLO-1 cells (4×10^6^ cells per animal) were injected in the right flank of four weeks-old female athymic nu/nu mice. Once the tumor reached 5 mm in size, mice were randomized in to one of the two groups, one treated with CDK 9 inhibitor and another treated with control vehicle (DMSO) with 5 mice in each group. The volume of xenograft tumor was measured every three days using a digital caliper. All mice were euthanized and tumor tissues were harvested 21 days after treatment. The tumor volume was calculated as (W^2^ x L)/2 (in which W=smallest diameter of tumor, L=largest diameter of tumor).

### Statistical analysis

*In vitro* experiments were repeated at least three times. For each assay Student's t-test was utilized to compare the difference between groups *in vitro* and xenograft data and CDK9 staining intensity in patient samples of Barrett's esophagus and esophageal adenocarcinoma. Errors are S.E values of averaged results. P value < 0.05 was considered as significant.

The study was performed under an approved institutional IRB with waiver of informed consent (LAB-04-0979, PI Maru) and IACUC (1155-RN00) protocols.

## SUPPLEMENTARY MATERIALS FIGURES


