# Supplementary material for: Antitumor effects of cyclin dependent kinase 9 inhibition in esophageal adenocarcinoma
Source: Oncotarget. 2017 Feb 23;8(17):28696–710. doi: 10.18632/oncotarget.15645 (PMC5438684; doi:10.18632/oncotarget.15645)
Supplement: Supplementary file 1 [file oncotarget-08-28696-s001.pdf]

Antitumor effects of cyclin dependent kinase 9 inhibition in esophageal adenocarcinoma

SUPPLEMENTARY FIGURES

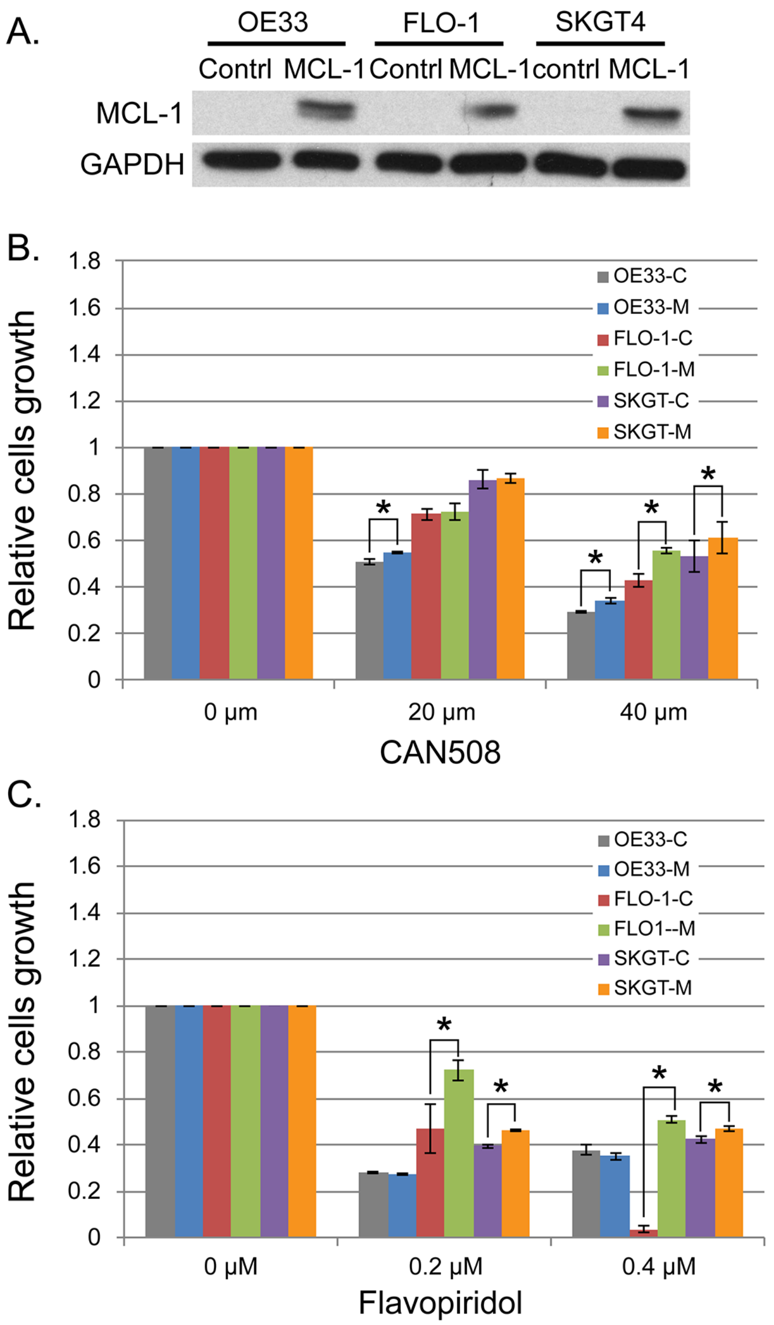

Supplementary Figure 1: The effect of MCL-1 on the sensitivity of esophageal adenocarcinoma cells to CDK9 inhibitors. (A) The overexpression of MCL-1 was confirmed by western blot. Overexpressing MCL-1 (OE-33-M, FLO-1-M, SKGT4-M) and control (OE-33-C, FLO-1-C, SKGT4-C) esophageal adenocarcinoma cells were treated with CAN508 for 72 hours **(B)** or Flavopiridol for 48 hours **(C)** at indicated doses. The proliferation of cells was measured by MTS assay using the CellTiter Aqueous One Solution Cell Proliferation Assay kit. Values are means ± SE of 3 independent experiments performed in triplicate. * represents p-value < 0.05 compared to control cells.

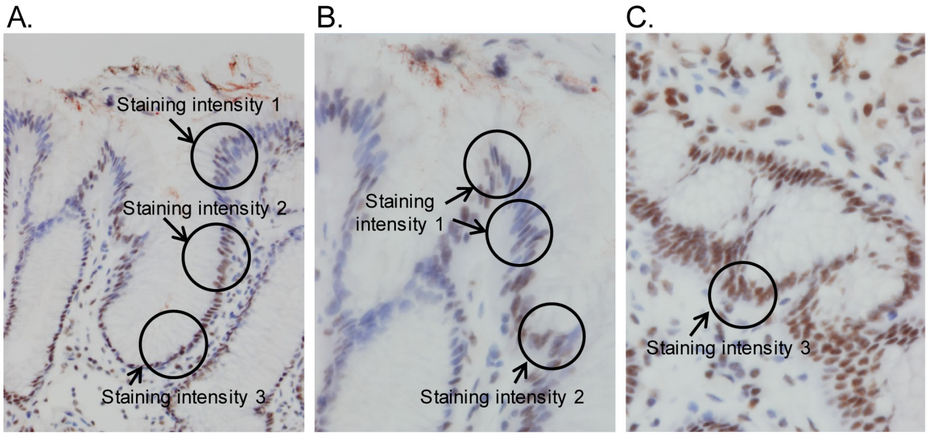

Supplementary Figure 2: Immunohistochemistry grading of intensity of CDK9 staining in columnar epithelium in tubular esophagus (Barrett`s esophagus). Supplementary Figure 1 A (200x) shows the entire crypt with staining intensity 1 and 2 in upper and middle part of the crypt and intensity 3 in the lower part of the crypt. Supplementary Figure 1B shows higher magnification (400X) of the upper part of crypt with intensity 1 and 2 staining and supplementary Figure 3 shows intensity 3 staining in the lower part of the crypt.
